# CDK7 in breast cancer: mechanisms of action and therapeutic potential

**DOI:** 10.1186/s12964-024-01577-y

**Published:** 2024-04-11

**Authors:** Ying Gong, Huiping Li

**Affiliations:** Key laboratory of Carcinogenesis and Translational Research (Ministry of Education/Beijing), Department of Breast Oncology, Peking University Cancer Hospital & Institute, Beijing 100142, China

**Keywords:** CDK7, Breast cancer, Mechanism, Targeted therapy, Small molecular inhibitor, Clinical trial

## Abstract

Cyclin-dependent kinase 7 (CDK7) serves as a pivotal regulator in orchestrating cellular cycle dynamics and gene transcriptional activity. Elevated expression levels of CDK7 have been ubiquitously documented across a spectrum of malignancies and have been concomitantly correlated with adverse clinical outcomes. This review delineates the biological roles of CDK7 and explicates the molecular pathways through which CDK7 exacerbates the oncogenic progression of breast cancer. Furthermore, we synthesize the extant literature to provide a comprehensive overview of the advancement of CDK7-specific small-molecule inhibitors, encapsulating both preclinical and clinical findings in breast cancer contexts. The accumulated evidence substantiates the conceptualization of CDK7 as a propitious therapeutic target in breast cancer management.

## Introduction

Breast cancer remains the most ubiquitous carcinoma worldwide, constituting approximately 30% of oncological diagnoses in females [1]. This pathology presents as an array of molecularly distinct subtypes, categorized by hormonal receptor and HER2 statuses—namely hormone receptor-positive (HR+), human epidermal growth factor receptor 2-positive (HER2+), and triple-negative breast cancer (TNBC). HR+/HER2- breast cancer, typified by the expression of estrogen and/or progesterone receptors and the lack of HER2 gene amplification, constitutes approximately 65–70% of all breast cancer cases [2]. As a central therapeutic approach for both early and late-stage manifestations of this disease, endocrine treatment focuses on strategies encompassing the reduction of estrogen synthesis, modulation of signaling through the ER, and antagonism or degradation of the ER itself [3]. The integration of CDK4/6 inhibitors with endocrine therapy has been sanctioned as a first-line approach in the metastatic setting. Three distinct orally administered CDK4/6 inhibitors, namely palbociclib, ribociclib, and abemaciclib, have been found to enhance progression-free survival (PFS) in conjunction with endocrine therapy. Nevertheless, extended administration of CDK4/6 inhibitors inevitably leads to drug resistance. TNBC, characterized by the absence of estrogen and progesterone receptors and HER2 overexpression, accounts for approximately 15% of all primary breast cancer diagnoses. TNBC represents the most virulent subtype of breast cancer, manifesting a markedly higher rate of recurrence and truncated overall survival. Increasingly, TNBC is recognized as a diverse and heterogeneous collection of diseases, the underlying biology of which remains enigmatic. This complexity has contributed to a lag in the development of targeted therapies for TNBC when compared to other breast cancer subtypes. In the current therapeutic landscape, cytotoxic chemotherapy endures as the cornerstone of standard care for this challenging category of breast cancer [4]. The advent of monoclonal antibodies, tyrosine kinase inhibitors, and antibody–drug conjugates targeting HER2 has markedly enhanced the therapeutic outcomes for HER2-positive breast cancer patients [5]. However, the clinical landscape is marred by instances of patient non-responsiveness or initial responsiveness followed by the eventual emergence of resistance.

Approximately 70–80% of patients diagnosed with early-stage, non-metastatic breast cancer experience curative outcomes [6]. In stark contrast, advanced breast cancer characterized by distant organ metastases remains a therapeutic conundrum, typically considered incurable with contemporary therapeutic strategies. Despite declining mortality rates attributed to advancements in early detection and innovative treatment regimens, contentious issues persist in the realms of therapeutic selection. These quandaries involve the formulation of optimal treatment paradigms for HR+/HER2- metastatic conditions that have developed resistance to conventional endocrine and CDK4/6 inhibitor regimens. Similarly, the challenges extend to identifying viable mechanisms for counteracting resistance to anti-HER2 interventions and optimizing the potency of immunotherapeutic approaches in TNBC. Addressing these clinical conundrums necessitates the identification of relevant biomarkers to augment a tailored treatment approach, thereby postponing the commencement of chemotherapy. A compelling candidate in this context is CDK7, a cyclin-dependent kinase-activating kinase that is integral to cell cycle progression and gene transcription [7]. Targeting CDK7 via various modalities, such as small interfering RNA (siRNA) or pharmacological inhibitors, has yielded encouraging antineoplastic results [8, 9]. Recent advancements have ushered in a cadre of CDK7 inhibitors into phase I/II clinical trials for breast cancer [10]. In this comprehensive review, we illuminate the multifaceted role of CDK7 in maintaining malignant phenotypes and fostering drug resistance across diverse molecular subtypes of breast cancer. Moreover, we provide an exhaustive overview of ongoing clinical trials targeting CDK7, corroborating its potential as a viable therapeutic target. Thus, our findings advocate for the strategic exploration of anti-CDK7 therapy as a promising avenue in breast cancer management.

## The biologic function of CDK7

**Fig. 1:**
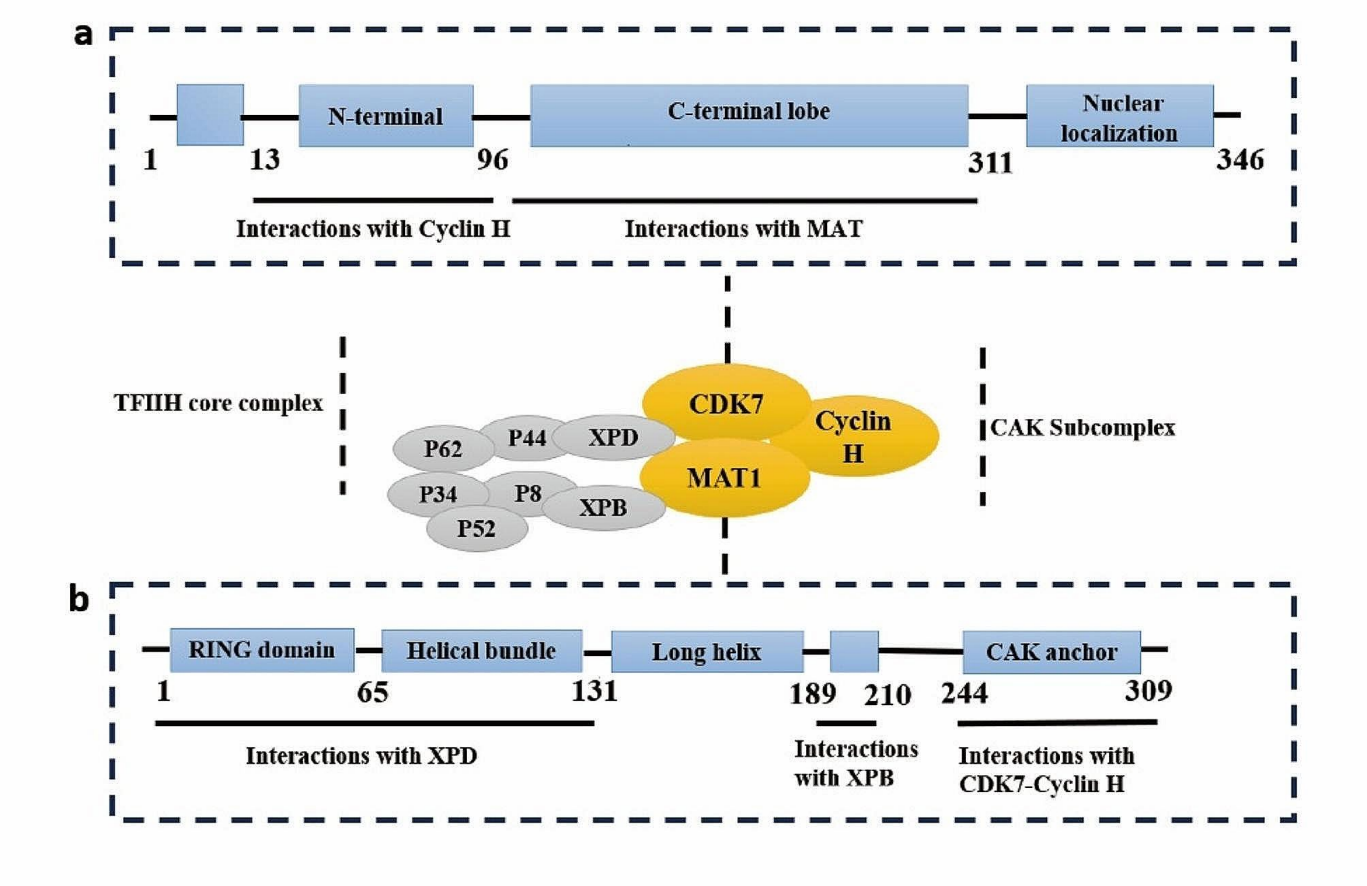
Structure of CAK and TFIIH. **(a)** CDK7, an enzyme comprising 346 amino acids. The N-terminal cyclin H-binding lobe (residues 13–96) primarily consists of β sheets and one α helix, while the C-terminal MAT1 binding lobe (residues 97–311) is predominantly composed of α helices. The N-terminal residues 1–12 and C-terminal residues 312–346 feature a putative nuclear localization sequence. **(b)** The structural of the MAT1 includes the RING domain and helical regions in the MAT1 N-terminus, and the CAK anchor in the MAT1 C-terminus.

**Fig. 2:**
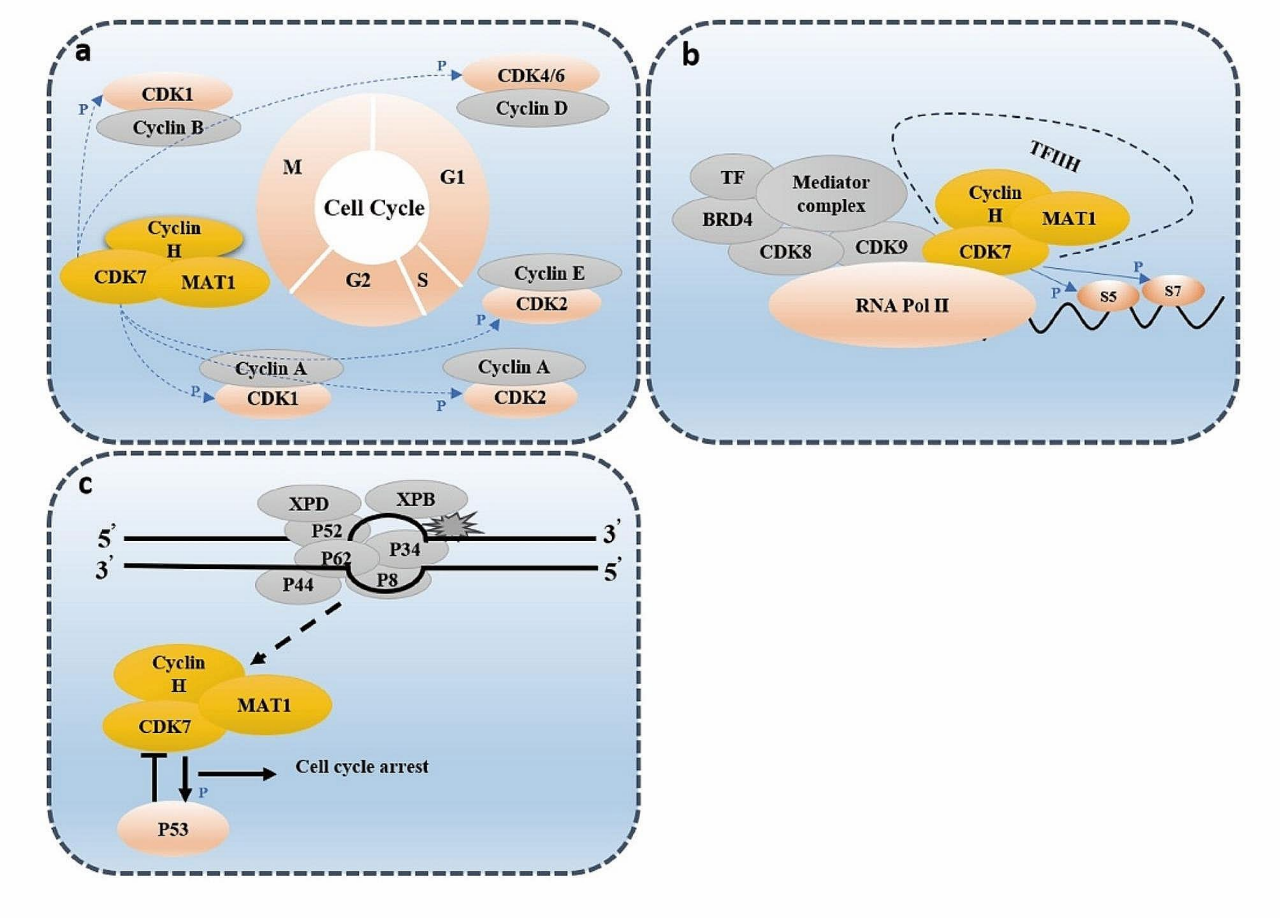
Role of CDK7 in regulating the cell cycle **(a)**, transcription **(b)** and DNA repair **(c)**. **(a)** The CAK complex phosphorylates the T-loop of key cell cycle CDKs, including CDK1, 2, 4, and 6. **(b)** As a component of TFIIH, CDK7 catalyzes the phosphorylation of Ser5 and Ser7 within the RNA Pol II C-terminal domain. **(c)** In DNA repair, the dislodgement of MAT1 from its binding sites on the TFIIH core complex results in the release of the 3-subunit CAK module. The CAK complex interact and the activate p53 through phosphorylating at Ser-33. In a reciprocal manner, p53 downregulates CAK kinase activity.

CDK7, a serine/threonine kinase composed of 346 amino acids, has a predicted molecular mass of 39 kDa. Structurally, CDK7 exhibits a classical kinase fold, comprising the N-terminal cyclin H-binding lobe (residues 13–96), primarily consisting of β sheets and one α helix, and a C-terminal MAT1 binding lobe (residues 97–311), predominantly composed of α helices. The N-terminal residues 1–12 and the C-terminal residues 312–346 encompass a putative nuclear localization sequence [11], as detailed in Fig. 1a. Within the cellular context, CDK7 forms a complex primarily with cyclin H and MAT1 to constitute the CDK-activating kinase (CAK) complex [12]. The CDK7 and cyclin H tandem establishes a canonical CDK-cyclin pair, while MAT1 assumes the role of a CAK assembly factor, heightening CAK activity toward target CDKs [13, 14]. Additionally, MAT1 serves to anchor the CAK to the core of general transcription factor IIH (TFIIH) (XPD, XPB, P8, P44, P62, P34, P52) during the transcription initiation process carried out by this 10-subunit complex [15, 16]. Notably, specific regions such as the RING domain and helical segments proximal to the MAT1 N-terminus, as well as the CAK anchor near the MAT1 C-terminus, engage with the TFIIH central complex and CDK7-cyclin H, respectively [17], as outlined in Fig. 1b.The CAK complex functions as a crucial promoter of cell cycle transition, achieving this by phosphorylating the T-loop of key cell cycle CDKs, including CDK1, 2, 4, and 6 (Fig. 2a). Essential to both the activation and complex formation of CDK1/cyclin-B during the G2-M phase transition, CDK7 is similarly vital for the activation of CDK2/cyclins in the G1-S phase transition [18–20]. Further, CDK7’s phosphorylation of CDK4/6 at Thr172 and Thr177 during the G1 phase initiates DNA synthesis, a process that responds to mitogen stimuli [20, 21]. In addition to its well-documented role in cell cycle regulation, CDK7 serves as an integral part in transcriptional regulation. As a component of the TFIIH, CDK7 catalyzes the phosphorylation of Ser5 and Ser7 within the RNA Pol II C-terminal domain (CTD) [22–24], an essential step in driving the clearance of the polymerase from the promoter. This phosphorylation event facilitates the release of RNA Pol II from the mediator, initiating transcription (Fig. 2b). Additionally, CDK7 phosphorylates CDK9, a core element of the positive transcription elongation factor b (P-TEFb), which subsequently phosphorylates the Ser2 residue of the RNA Pol II CTD, thereby promoting transcription elongation [25]. The regulatory landscape is further complexified with the response to DNA damage. During DNA repair, the initial recognition of damage is orchestrated by XPC-RAD23B, followed by the recruitment of TFIIH. Upon TFIIH loading, XPB initiates the unwinding of the DNA, enabling TFIIH to tilt against the DNA and positioning XPD to bind the damaged strand 5’ to the lesion. The binding event is closely monitored by MAT1, which signals to CAK to disengage from TFIIH, thereby allowing the progression of the repair process [26, 27]. Following the completion of DNA repair, CAK reassociates with TFIIH, and the complex resumes its transcriptional role. The tumor suppressor protein p53, recognized as a transcriptional activator, plays a pivotal role in mediating cellular responses to DNA damage by inducing apoptosis and cell cycle arrest. The CAK complex interacts with p53 and activates it through phosphorylation at Ser-33. In a reciprocal manner, p53 downregulates CAK kinase activity [28, 29]. This intricate feedback mechanism may lead to a cessation of both the cell cycle and transcription, thereby facilitating cell recovery or triggering apoptosis, as depicted in Fig. 2c. Thus, CDK7 emerges as a multifaceted regulatory protein, involved in cell cycle control, RNA polymerase II-mediated RNA transcription, and DNA repair processes.

### CDK7 in cancer

CDK7’s vital role in the preservation of malignant phenotypes across various cancer cells is underscored by substantial evidence [30]. Comparative analysis reveals elevated CDK7 expression in tumor tissues across a spectrum of neoplasms, including but not limited to breast. Notably, increased CDK7 expression correlates with a dismal prognosis [31]. Targeting CDK7 has revealed pronounced effects on cancer cell proliferation, migration, invasion, stemness, and drug resistance across malignancies such as breast cancer [32], lung cancer [33], hepatocellular carcinoma [34], thyroid cancer [35], glioblastoma [36], gastric cancer [37], pancreatic cancer [38], gallbladder cancer [39], colorectal cancer [40], osteosarcoma [41], lymphomas [42], leukemia [43], among others. In recent years, a range of specific small molecular inhibitors targeting CDK7 has been synthesized and classified, as detailed in Table 1. The set encompasses reversible ATP-binding site inhibitors such as BS-181 [44], CT7001 [45], LDC4297 [46], QS1189 [42], SY-5609 [47]. Additionally, the development of ATP-competitive covalent inhibitors has further expanded this field, with notable examples including THZ1 [48], THZ2 [49], SY-1365 [50], YKL-5-124 [51], and YKL-1-116 [52]. The specific CDK7 small molecular inhibitors have yielded promising results in suppressing malignant phenotypes across over 23 cancer types, especially in breast cancer. Importantly, nine specific CDK7 inhibitors, CT7001, SY5609, Q901, SY1365, XL102, TY-2699a, GTAEXS-617, EOC237, LY3405105, have now have reached Phase I/II clinical trials, reflecting an evolving therapeutic landscape (Table 1).

**Table 1:** Specific CDK7 inhibitors under development

Name	CAS No.	Fomula	Preclinical tests	Highest Clinical Phase
BS-181	1092443-52-1	C_22_H_32_N_6_	Breast cancer [70], Gastric cancer [54], Osteosarcoma [115], Synovial sarcoma [116], Thyroid carcinoma [117], T cell acute lymphoblastic leukemia [118]	No studies entered clinical studies yet
CT7001	1805833-75-3	C_22_H_30_N_6_O	Breast cancer [45], Prostate cancer [119]	Phase 1/2
LDC4297	1453834-21-3	C_23_H_28_N_8_O	Breast cancer [93], Pancreatic cancer [38], Lung cancer [120]	No studies entered clinical studies yet
QS1189	-	-	Mantle cell lymphoma [42]	No studies entered clinical studies yet
THZ1	1604810-83-4	C_31_H_28_C_l_N_7_O_2_	Breast cancer [95], Head and neck squamous cell carcinoma[121], Thyroid cancer [122], Neuroblastoma [123], Glioblastoma [124], Gastrointestinal stromal tumours [125], Pancreatic cancer [126], Hepatocellular carcinoma [127], Gallbladder cancer [39], Cholangiocarcinoma [128], Colorectal cancer [40], Nasopharyngeal carcinoma [129], Prostate cancer [130], Renal cell carcinoma [131], Urothelial carcinoma [132], Bladder cancer [133], Melanoma [134], Osteosarcoma [135], lymphomas [62], Leukemia [43]	No studies entered clinical studies yet
THZ2	1604810-84-5	C_31_H_28_C_l_N_7_O_2_	Osteosarcoma [49], Gastric cancer [136]	No studies entered clinical studies yet
YKL-5-124	1957203-01-8	C_28_H_33_N_7_O_3_	Lung cancer [53], Myeloma [137], Testicular germ cell tumors [138], Neuroblastoma [139]	No studies entered clinical studies yet
SY 5609	2417302-07-7	C_23_H_26_F_3_N_6_OP	Breast cancer [47], Ovarian cancer [10]	Phase 1
Q901	2379284-04-3	C_33_H_36_FN_9_O_2_	-	Phase 1/2
SY1365	1816989-16-8	C_31_H_35_C_l_N_8_O_2_	Breast cancer, Ovarian cancer, Acute myelocytic leukemia [50]	Phase 1
XL102	-	-	-	Phase 1
YKL-1-116	1957202-71-9	C_34_H_38_N_8_O_3_	Colon cancer [140]	No studies entered clinical studies yet
TY-2699a	-	-	-	Phase 1
EOC237	-	-	-	Phase 1
GTAEXS-617	-	-	-	Phase 1/2
LY3405105	2326428-25-3	C_26_H_39_N_7_O_3_	Breast cancer, Ovarian cancer, Lung cancer, Gastric cancer, Colon cancer [141]	Phase 1

Investigations into the molecular mechanisms underlying anti-tumor effects of targeting CDK7 have primarily implicated its role in cell cycle arrest and the suppression of CDK7-dependent gene transcription [53]. CDK7 suppression with inhibitors or siRNA fosters apoptosis, increasing the G2/M cell population [54–56] and shRNA-mediated silencing of CDK7 leads to an impairment in T-loop phosphorylation of CDKs, thereby arresting the cell cycle and impeding cell proliferation [57]. Clusters of transcriptional enhancers driving expression of genes that define cell identity have been defined super-enhancers (SEs) [58]. The intricate interplay between transcriptional regulators, including CDK7, and SEs is further highlighted by the observation that cancer cells exhibit transcriptional addiction, demanding higher transcription levels to maintain growth [59]. Consequently, the targeting of transcription regulators like CDK7 has emerged as a strategy to dismantle this transcription boost [60], with THZ1 and THZ2 showing particular efficacy against cancers that rely on SE-driven genes including *C-MYC*, *RUNX2*, *STAT*, *SOX2*, *MITF*, *SOX9* [32, 33, 48, 49, 61–64]. Recently, our team revealed a new function of CDK7 in mediating metabolic process of esophageal cancer. We discovered that CDK7 phosphorylated YAP at S127 and S397 sites in the nucleus and enhanced D-lactate dehydrogenase protein expression, therefore helping esophageal cancer stem cells escape from ferroptosis [65]. Overall, these findings underscore CDK7 as a critical mediator in cancer pathogenesis and opens avenues for targeted therapeutic interventions, thus heralding a new frontier in cancer research.

### The expression of CDK7 and its prognostic value in breast cancer

In an examination of CDK7’s abundance within normal breast epithelium and a cohort of 12 breast cancer cell lines, immunoblotting analysis distinctly revealed elevated CDK7 protein expression within malignant counterparts as opposed to normal breast epithelium [66]. This observation was further corroborated through a comprehensive analysis of CDK7 mRNA levels within breast cancer patients, utilizing an array of public databases including METABRIC microarray [67], Oncomine, GEPIA2, Human Protein Atlas (HPA) database [68], and the GENT database [69]. Moreover, a detailed investigation based on a tissue array encompassing 140 patients and an expansive dataset exceeding 4,000 samples sourced from the bc-GenExMiner database provided additional insights. It highlighted that CDK7 protein levels were significantly higher within HR-positive and HER2-positive breast cancer subtypes, as contrasted with the TNBC subtype [69].

The prognostic significance of CDK7 in breast cancer remains a matter of considerable debate. One study, utilizing publicly available transcriptomic data from a specific group of TNBC patients (*n* = 383) and the METABRIC TNBC dataset (*n* = 217), identified that elevated CDK7 mRNA levels were synonymous with reduced relapse free survival (RFS) and poor breast cancer specific survival (BCSS). This association extended to higher CDK7 protein expression and unfavorable prognosis within the RATHER TNBC TMA cohort (*n* = 109) and METABRIC TNBC TMA cohort (*n* = 203) [70]. However, in an unstratified BreastMark and METABRIC cohort, no significant correlation between CDK7 expression and prognosis was observed [70], leading to the hypothesis that CDK7 might serve as a predictive factor specifically within the TNBC subgroup, rather than across breast cancer in general.

Contrarily, another study demonstrated that elevated CDK7 expression correlated with worse RFS across a broad and unselected cohort of breast cancer patients (*n* = 3,951), representing multiple subtypes [71]. This observation was further strengthened within specific subtypes, including HR+/HER2-, HER2+ breast cancer and TNBC, where higher CDK7 mRNA levels were associated with diminished RFS [71]. Such findings intimate that CDK7 could serve as a universal target for breast cancer treatment, irrespective of subtype. In alignment with the latter study, additional research utilizing platforms such as UALCAN, TCGA portal, and Kaplan-Meier Plotter underscored the connection between elevated CDK7 mRNA expression and poorer RFS and overall survival (OS) in breast cancer patients [68, 69]. Remarkably, another study presented an opposing viewpoint, revealing that increased CDK7 protein levels were indicative of a more favorable prognosis. Analyzing breast cancer TMAs for CDK7 (*n* = 945), the study found that patients with high-grade and extensive tumors, or those with recurrent disease, exhibited lower CDK7 expression. Elevated CDK7 levels were instead associated with extended BCSS and prolonged time to distant metastasis [67].

This landscape of disparate findings illustrates the complexity of CDK7’s role within breast cancer prognosis and highlights the necessity for further refined and targeted investigations. An essential avenue for future exploration involves investigating whether the efficacy of CDK7 targeting therapy is contingent upon the expression level of CDK7. Furthermore, the question arises as to whether CDK7 expression should be routinely assessed in the treatment of advanced breast cancer with CDK7 small-molecule inhibitors.

### CDK7 in ER-positive breast cancer

Analysis of TCGA ER-positive (ER+) breast cancer samples has revealed a positive correlation between CDK7 and ER mRNA levels, and high CDK7 expression has been associated with notably shorter OS for ER+ breast cancer patients [72]. Experimental approaches targeting CDK7 through specific small molecular inhibitors, such as THZ1 [72], LDC4297 [73], ICEC0942 [45], SNS-032 [74], and roscovitine [75], have proven effective in suppressing ER+ breast cancer cells. Specifically, these treatments have been demonstrated to inhibit the proliferation of ER+ breast cancer cells, induce apoptosis, and suppress the growth of breast cancer xenografts in murine models [45, 72–74]. Most remarkably, the combination of CDK7 inhibitors with known therapeutic agents like fulvestrant [45] or tamoxifen [72, 75] has shown enhanced efficacy in inhibiting tumor growth compared to either single agent, highlighting the potential therapeutic advantage of these combinations.

Mechanisms of endocrine treatment resistance have been described in previous studies. ER becomes an activated transcription factor upon estrogen binding and dimerization. A positive correlation has been identified between the expression of pERα-S118 and poorer disease-free and overall survival in HR+/HER2- breast cancer patients, as well as resistance to tamoxifen [76, 77]. Studies indicate that CDK7 activates ER phosphorylation at Ser118, enhancing MYC transcription and mediating tamoxifen resistance in ER+ breast cancer [72]. Targeting CDK7 via inhibitors like THZ1 or roscovitine has been found to abolish this phosphorylation, thereby preventing ER activation and the expression of target genes such as *MYC* [78, 79]. Specifically, mutations in the ER result in constitutive ER activity even in the absence of estrogen, thereby altering the transcriptional dynamics of the ER pathway and contributing to the complex landscape of endocrine resistance [79–82]. Interestingly, THZ1 treatment effectively prevented ER mutants (e.g., Y537S, Y537N, and D538G) phosphorylation in MCF7 and T47D cells, and the key pathways that inhibited by THZ1 were related to the ErbB/PI3K/mTOR pathway [83], suggesting that THZ1 is targeting the ER mutant transcriptional network. In addition, emerging research highlights estrogen treatment increases tumorsphere formation capacity, and tamoxifen-resistant cell lines exhibit higher number of cancer stem cell (CSC) populations [84, 85]. Targeting CDK7 has proven effective in inhibiting tumorsphere formation and CSC biomarkers in MCF-7 and LCC2 cells [86]. Previous studies confirm mutation in PIK3CA-AKT1-mTORC pathway is the main factor mediating resistance of CDK4/6 targeted therapy [87]. Recently, a marked increase in the expression of CDK7 and other kinases, has been observed in palbociclib-resistant MCF-7 cells. Intriguingly, THZ1 could effectively inhibit the survival of palbociclib-resistant cell lines [88]. These findings further suggesting the therapeutic potential of CDK7 targeting in reversing drug resistance of endocrine or CDK4/6 targeted therapy in HR+/HER2- breast cancer. Based on the comprehensive results of the above studies, we can observe that CDK7 inhibitors are not only effective as monotherapy but also exhibit efficacy in combination with endocrine therapy. They are effective not only against endocrine-sensitive ER+ cells but also against ER+ cells that are resistant to both endocrine and CDK4/6 inhibitors. Therefore, in the future, it is necessary to clarify the timing of CDK7 inhibitor application—whether it should be used in combination with endocrine therapy from the outset or deferred until resistance develops.

Subsequent investigations have been conducted to elucidate the underlying mechanisms of acquired resistance to CDK7 inhibitors, ICEC0942 and THZ1, within the context of breast cancer treatment. Resistant MCF7 cell lines were synthetized via extended culture in the presence of either ICEC0942 or THZ1. Detailed analysis revealed that ABCB1 upregulation could instigate resistance to both ICEC0942 and THZ1, whereas upregulation of ABCG2 resulted in specific resistance to THZ1, while maintaining sensitivity to ICEC0942. The resistance could be counteracted by employing a competitive inhibitor of ABCB1, verapamil, or a non-competitive inhibitor, tariquidar, thereby restoring cellular responsiveness to both drugs [89]. In a related development, functional enhancement of p53 was discovered to augment the sensitivity of MCF-7 cells to THZ1 treatment. The synergistic use of THZ1 in combination with nutlin-3 or alternative strategies to boost p53 expression facilitated a considerable enhancement in the anticancer efficacy of THZ1, resulting in significant breast cancer cell lethality [90].

### CDK7 in triple-negative breast cancer

A series of investigations have shed light on the potential of targeting CDK7 in the treatment of TNBC. Utilizing small molecular inhibitors such as THZ1 [32, 70, 91], BS-181 [92], LDC4297 [93], and N76-1 [94], researchers have effectively inhibited the malignant phenotypes of TNBC cells. Interestingly, TNBC cells displayed heightened sensitivity to CDK7 inhibition when compared to HR+ breast cancer cells [93]. Therapeutic application of THZ1 was further shown to inhibit patient-derived xenografts (PDXs) of TNBC [32], underscoring the potential of CDK7 inhibition as a promising avenue for the management of this aggressive cancer subtype. Emerging research has unveiled a phenomenon of “transcriptional addiction” within cancer cells, supporting their uncontrolled proliferation and other functional needs. Tumor cells were found to be highly reliant on transcriptional machinery [59], with CDK7 mediating this addiction to a critical cluster of genes in TNBC. Genes such as *SOX9*, *MYC*, *FOXC1*, *EGFR*, and *FOSL1*, essential for TNBC tumorigenicity and frequently associated with super-enhancers, demonstrated particular sensitivity to CDK7 inhibition [32]. Consistently, transcription factors including SOX9 [95], FOXC1, MYC [96], and KLF5 [91] were observed to promote cell proliferation, migration, and stemness in TNBC cells. Elevated expression levels of these genes corresponded with poor prognosis in TNBC patients. Additionally, CDK7 was instrumental in maintaining chromatin sublooping, mediating the transcription of several condensin subunits, such as structural maintenance of chromosomes 2. Inhibition of CDK7 led to the prolongation of mitosis and induced chromatin bridge formation, DNA double-strand breaks, and abnormal nuclear features. Notably, the regulation of condensin subunit gene expression by CDK7 was found to be independent of super-enhancers [97].

Immunotherapy has been observed to confer significant benefits to patients with TNBC, outperforming other breast cancer subtypes in therapeutic efficacy. Clinically, the presence of programmed death ligand 1 (PD-L1) or programmed death 1 (PD-1) within the tumor microenvironment has been identified as a marker for predicting responsiveness to immunotherapy in TNBC patients [98]. The KEYNOTE-522 and KEYNOTE-355 trials have collectively demonstrated that the integration of the PD-1 inhibitor pembrolizumab with standard chemotherapy augments pathological complete remission rates or OS in early-stage or metastatic TNBC patients when compared to chemotherapy alone [4]. A preclinical investigation revealed that a combination of THZ1 with an anti-PD-L1 antibody surpassed the single administration group in terms of inhibiting tumor growth within a TNBC xenograft mouse model [96]. In a separate innovation, EGFR-targeted chimeric antigen receptor (CAR) T cells have been proven potent and specific in suppressing TNBC growth both in vitro and in vivo [99, 100]. Despite initial success, a subset of mice promptly developed resistance. This resistance was mitigated through a combination therapy involving THZ1 and EGFR CAR T cells, which suppressed immune resistance in human MDA-MB-231 cell-derived and TNBC patient-derived xenografts by inhibiting the expression of CAR T-cell-induced immunosuppressive genes [101]. Consequently, the exploration of CDK7 inhibitors as immunoregulators has garnered attention. However, the compelling immunomodulatory potential inherent in CDK7’s role in the anti-tumor immune response is presently in its nascent stages. A comprehensive understanding of the direct impacts of CDK7 inhibition on both stromal and immune cells is imperative for nuanced analysis within the framework of breast cancers.

In addition to its pivotal role in orchestrating transcriptional machinery, CDK7 has been identified as having a direct oncogenic function through protein-protein interaction. Specifically, CDK7 interacts with C-terminal binding protein 2 (CtBP2), a protein known to promote tumor progression by enhancing epithelial-mesenchymal transition (EMT) and inhibiting apoptosis in cancer cells. Within this complex, CDK7 competes with the tumor repressor HIPK2 for CtBP2 binding. This interaction consequently inhibits the phosphorylation and dimerization of CtBP2, leading to its degradation in MDA-MB-231 cells. Thus, CDK7 serves to stabilize CtBP2 positively. The novel insights into the CDK7-CtBP2 axis provide a foundation for exploring this pathway as a potential anti-tumor strategy in TNBC [102].

The cumulative evidence from preclinical and clinical investigations underscores the potential of CDK7 inhibition as a promising strategy for managing TNBC. However, a significant obstacle to this approach is the likelihood of recurrent disease stemming from acquired resistance. Recognizing and targeting the underlying mechanisms of CDK7 inhibitor resistance will be instrumental in the forward development of therapies capable of bypassing this resistance. Recent research has revealed that both ABCG2 and phosphorylation of SMAD3 were upregulated in THZ1-resistant MDA-MB-468 cells. By employing either siRNA or small molecular inhibitors to suppress ABCG2 or SMAD4 (a transcriptional partner of SMAD3), researchers were able to resensitize resistant cells to CDK7 inhibitor [103]. These findings unveil the TGF-β/Activin-ABCG2 pathway as a potential target for both preventing and overcoming resistance to CDK7 inhibitors, thereby paving the way for further exploration in the context of TNBC treatment.

### CDK7 in HER2-positive breast cancer

Numerous intrinsic or acquired resistance mechanisms to HER2-targeted therapy have been delineated, including mutations within the HER family of receptors that induce activation of downstream signaling cascades such as the PI3K-AKT and RAS-MAPK pathways, and the overactivation of compensatory elements like CDK2 and Cyclin D1 [104]. Such a complex matrix of resistance underlines the urgent necessity for innovative treatments targeting aberrant kinome activation that underpins resistance to HER2-targeted therapy. Intriguingly, recent investigations have illuminated the role of CDK7, whose expression is augmented by AKT in human HER2 inhibitor-resistant breast cancer cells. Functioning as a transcriptional cofactor, CDK7 induces aberrant gene upregulation that facilitates resistance to HER2 inhibition. Demonstrated both in vitro and in vivo, THZ1 has exhibited potent synergistic effects with the HER2 inhibitor lapatinib in HER2 inhibitor-resistant breast cancer cells [105]. This body of evidence fortifies the conceptualization of CDK7 inhibition as a viable additional therapeutic pathway, specifically targeting the activation of genes involved in the resistance fostered by multiple HER2 inhibitor-resistant kinases.

### The specific CDK7 inhibitors in breast cancer clinical trails

A suite of oral specific CDK7 inhibitors, including SY5609, CT7001, XL102, TY-2699, GTAEXS-617 and intravenous specific CDK7 inhibitors such as Q901, SY1365, have made strides into phase I/II breast clinical trials (Table 2). As shown in Table 2, the specific CDK7 inhibitors are predominantly assessed in patients with locally advanced or metastatic HR+/HER2- breast cancer, who exhibited failure in prior treatment with a CDK4/6 inhibitor in combination with hormonal therapy. Recently, the results of a clinical trial (ClinicalTrials.gov: NCT03363893) evaluating the safety and efficacy of the CT7001 in advanced breast cancer were published [106]. The trial determined a maximum tolerated dose of CT7001 to be 360 mg once daily, with a half-life of approximately 75 hours. The most common adverse events were low-grade nausea, vomiting, and diarrhea. Among the evaluated patients, one partial response (PR) (duration 337 days) and a clinical benefit rate at 24 weeks (CBR) of 20.0% (4/20) were achieved in locally advanced and/or metastatic TNBC patients administered CT7001 at 360 mg once daily. A cohort of 31 patients with post-CDK4/6 inhibitor HR+/HER2- advanced breast cancer received CT7001 in combination with fulvestrant, resulting in 3 patients achieving PR with a CBR of 36.0% (9/25) [106]. These findings underscore the therapeutic potential of CT7001, particularly in addressing the unmet medical needs of patients whose disease has progressed on CDK4/6 inhibitors.

**Table 2:** Specific CDK7 inhibitors in breast cancer clinical trials

Name	NCT Number	Clinical Phase	Status	Conditions	Administration route	Interventions
SY5609	NCT04247126	Phase 1	Completed	Advanced or metastatic HR+/HER2- breast cancer who failed prior treatment with a CDK4/6 inhibitor in combination with hormonal therapy in a previous line of therapy	Orally	Drug:SY5609 Drug: Fulvestrant
Q901	NCT05394103	Phase 1/2	Recruiting	Advanced or metastatic HR+/HER2- breast cancer, who have progressed following standard-of-care therapy	Intravenous infusion	Drug: Q901
CT7001	NCT03363893	Phase 1/2	Completed	Locally advanced or metastatic TNBC, HR+ /HER2- breast cancer	Orally	Drug: CT7001 Drug: CT7001 in combination with fulvestrant
SY1365	NCT03134638	Phase 1	Terminated	Advanced or metastatic HR+/HER2- breast cancer who failed prior treatment with a CDK4/6 inhibitor in combination with hormonal therapy in a previous line of therapy	Intravenous infusion	Drug:SY1365 Drug: Fulvestrant
XL102	NCT04726332	Phase 1	Recruiting	Advanced or metastatic HR+/HER2- breast cancer, TNBC, who have progressed following standard-of-care therapy	Orally	Drug: XL102 Drug: Fulvestrant
TY-2699a	NCT05866692	Phase 1	Recruiting	Locally advanced or metastatic TNBC, HR+/HER2- breast cancer	Orally	Drug: TY-2699a
GTAEXS-617	NCT05985655	Phase 1/2	Recruiting	Advanced breast carcinoma (HR+/HER2- that has progressed to a prior treatment with CDK4/6 inhibitor)	Orally	Drug: GTAEXS-617

## Discussion and conclusion

CDK7, a pivotal component of theCAK complex, assumes a dual functional role. Firstly, it phosphorylates various CDKs, thereby facilitating the progression of the cell cycle. Additionally, CDK7 targets the CTD of RNA Polymerase II, a crucial process in gene transcription. Notably, certain investigations have indicated that CDK7 phosphorylates Ser5 and Ser7 within the CTD. However, it is worth noting that the inhibition of CDK7 using YKL-5-124, which apparently exhibits improved selectivity, does not effectively suppress the expression of Ser5 and Ser7 of CTD, as indicated in specific investigations [51]. Interestingly, murine embryonic fibroblasts lacking CDK7 expression demonstrate unaltered Pol II CTD Ser5 phosphorylation and a primarily unchanged gene expression program [57]. These results challenge the conventional understanding of CDK7 as the CTD kinase and consequently as a transcriptional regulator. It is plausible that both CDK7 and another CDK could fulfill the role of the CTD Ser5 kinase. In light of this possibility, it would be worthwhile to individually or in combination deplete other potential CDKs using CRISPR-Cas9 to determine which ones could complement the specific CDK7 inhibitors in blocking CTD Ser5 phosphorylation [107].

**Fig. 3:**
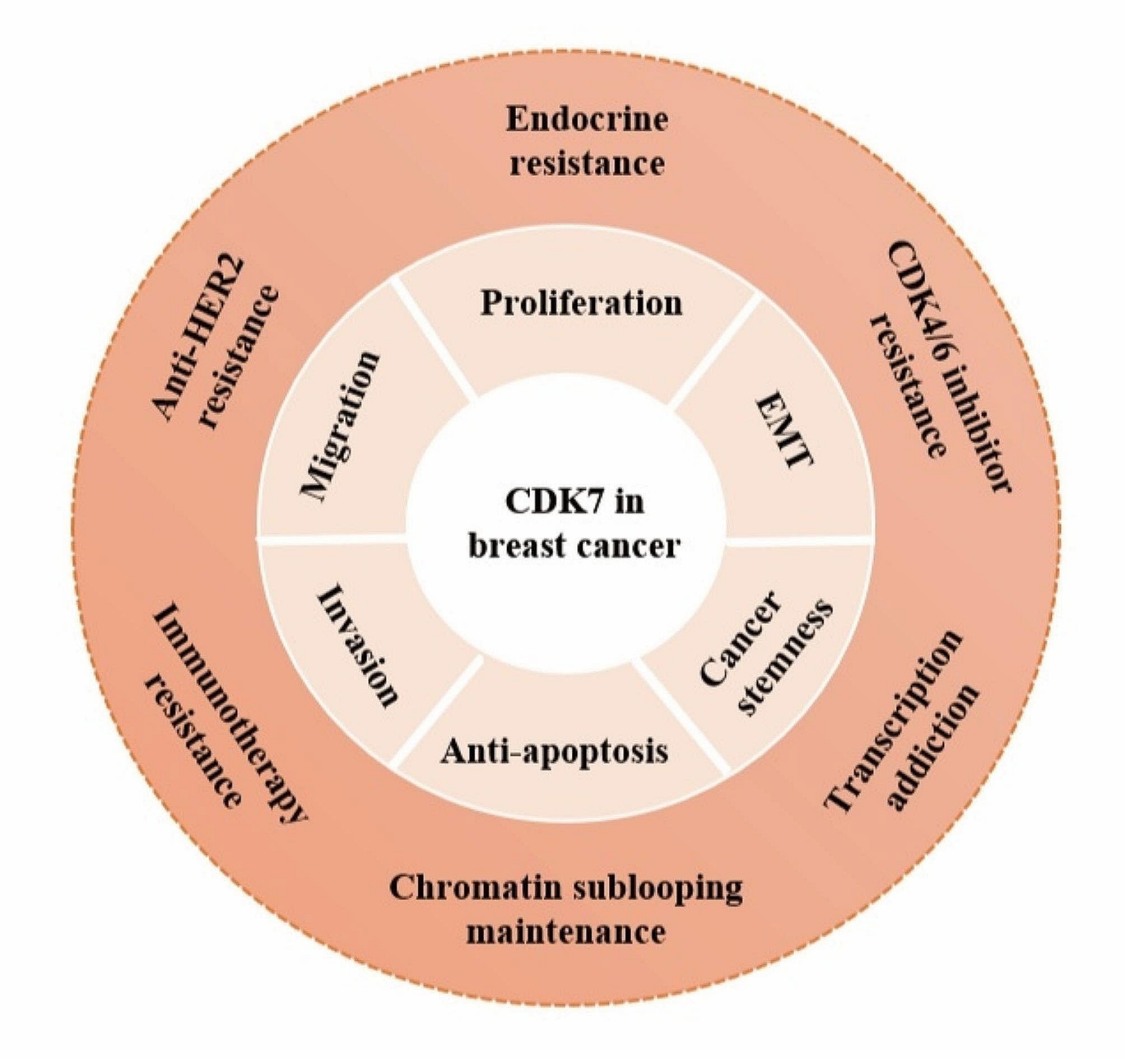
A pattern diagram of CDK7 in breast cancer

In this manuscript, we provide a comprehensive overview of the contemporary research landscape surrounding CDK7 within the domain of breast cancer. As delineated in Fig. 3, the intricate involvement of CDK7 is underscored in fostering the processes of proliferation, invasion, migration, and resistance to apoptosis in breast cancer cells. Further mechanisms studies found inhibiting CDK7 impaired the abnormal phosphorylation of ER, weekend the self-renewal ability of cancer stem cells, and reversed endocrine drug resistance in ER+ breast cancer cells. Current investigations predominantly focus on HR-positive and triple-negative breast cancer, with scant data on HER2-positive variants. Only one research showed that CDK7 inhibitor THZ1 exhibited potent synergistic effects with the HER2 inhibitor lapatinib in HER2 inhibitor-resistant breast cancer cells. Due to the lack of effective biomarkers, the development and appliance of targeted therapy is limited in TNBC. Inhibiting CDK7 suppressed the survival of triple negative breast cancer cells and patient-derived xenografts. CDK7 has been implicated in sustaining malignant phenotypes by mediating transcriptional addiction dependent of super-enhancers, maintaining chromatin sublooping independent of super-enhancers, and inhibiting the degradation of oncogenic protein in a manner of protein-protein interaction. Moreover, preliminary findings suggest that CDK7 inhibitors could potentiate immunotherapy responses, thereby laying the groundwork for future therapeutic strategies.

Evolutionary studies suggest that CDKs are divided into cell-cycle-related CDKs such as CDK1, CDK2, CDK4 and CDK6 and transcriptional CDKs including CDK7, CDK8, CDK9 and CDK11 [108, 109]. The recent FDA endorsement of CDK4/6 inhibitors for managing HR+/HER2- breast cancer indicates the burgeoning interest in CDK-targeted therapies. However, an established continuum of care remains elusive for patients with advanced HR+/HER2− breast cancer experiencing disease progression following CDK4/6 inhibition. Present therapeutic strategies gravitate toward mTOR inhibition with everolimus and PI3K inhibition with alpelisib. Insight from the phase I/II TRINITI study, involving a regimen of exemestane, ribociclib, and everolimus, revealed a CBR of 41% at week 24 and a median PFS of 5.7 months in a population where 92% had prior CDK4/6 inhibitor exposure [110]. Alpelisib’s application, albeit potent, is confined to patients harboring a PIK3CA mutation, representing approximately 40% of the cohort, achieving a median PFS of 7.3 months post-CDK4/6 inhibition, with a significant caveat of induced hyperglycemia necessitating anti-diabetic intervention [111]. Furthermore, preliminary data from DESTINY-Breast04 [112] and TROPiCS-02 [113, 114] trials highlight the potential of Trastuzumab deruxtecan and Sacituzumab govitecan, the HER2-targeted antibody-drug conjugate, delineating a novel therapeutic avenue for patients with advanced HR+/HER2− breast cancer following CDK4/6 inhibitor treatment. The specific CDK7 inhibitor, CT7001, in conjunction with fulvestrant, demonstrates a competitive clinical benefit rate (CBR of 39%) and acceptable adverse effects in advanced HR+/HER2− breast cancer patients. Notably, preclinical and clinical evidence underscores the utmost promise of targeting CDK7 in the subset of advanced HR+/HER2− breast cancer cases that have experienced disease progression following prior treatment with a CDK4/6 inhibitor in combination with hormonal therapy. Despite these encouraging findings, it is imperative to acknowledge the limited availability of only phase I/II clinical trial data, necessitating the imperative acquisition of phase III clinical data to affirm the safety and efficacy of CDK7 inhibitors in future clinical practice. Furthermore, pivotal head-to-head comparative trials are indispensable for the comprehensive evaluation of the efficacy of CDK7 inhibitors in treating advanced HR+/HER2− breast cancer in relation to established therapeutic modalities such as everolimus, alpelisib, sacituzumab govitecan, and others. Such comparative analyses aim to delineate the optimal management paradigm for this patient cohort. Additionally, there exists a compelling need for further investigations to ascertain the most promising combination strategies for CDK7 inhibitor therapy. Evolutionary insights, as gleaned from phylogenetic analyses, illuminate the structural similarity between CDK family members. Given the high structural resemblance among subtypes, CDK7 blockers inherently exhibit off-target activity towards other isoforms, thereby engendering potential risks of side effects. Notably, the CDK7 covalent inhibitor THZ1 exemplifies this phenomenon by displaying inhibitory activity on other kinases, including CDK12/13. Consequently, the termination of the clinical trial of the CDK7 covalent inhibitor SY-1365 underscores the challenges associated with achieving both efficient anti-tumor effects and safety. The ongoing discourse hinges upon the advantages conferred by highly selective inhibitors that may concurrently exhibit some degree of off-target activity, prompting a nuanced consideration in the continued development of CDK7 inhibitors.

## Abbreviations

CAR-T cells: chimeric antigen receptor T cells
CAK: CDK-activating kinase
CBR: clinical benefit rate
CDK7: Cyclin-dependent kinase 7
HR: hormone receptor
HER2: human epidermal growth factor receptor 2
NER: nucleotide excision repair OS,overall survival
P-TEFb: positive transcription elongation factor b
PDX: patient-derived xenograft
PD-L1: programmed death ligand 1
PD-1: programmed death 1
PFS: progression-free survival
PR: partial response
siRNA: small interfering RNA
TNBC: triple-negative breast cancer
TFIIH: general transcription factor IIH
